# Gyroid porous inserts as a novel flow control to mitigate tip vortex cavitation

**DOI:** 10.1007/s00348-026-04226-x

**Published:** 2026-05-23

**Authors:** Thomas Berger, Mohamed Farhat

**Affiliations:** Institute of Mechanical Engineering, École Polytechnique Fédérale de Lausanne, Avenue de Cour 33 Bis, 1007 Lausanne, Switzerland

## Abstract

Tip vortices generated at the tip of lifting surfaces pose significant challenges in fluid dynamics, causing induced drag, noise, and cavitation erosion risk across aerospace and hydraulic applications. Among the various mitigation strategies, porous tips have been explored with mixed results, showing limited effectiveness in diffusing concentrated vorticity. In this study, we introduce a gyroid-based porous tip as a novel passive flow control device for tip vortex mitigation. A gyroid is a triply periodic minimal surface that forms a smooth and tortuous porous 3D network. A gyroid-based porous insert was attached to an elliptical NACA 16-020 hydrofoil tip (Re ≈ 9 × 10^5^), replacing 3%, 5%, and 9% of the span. Laser Doppler Velocimetry (LDV) measurements revealed that increasing the gyroid portion dramatically reduces maximum tangential velocity while enlarging the vortex core radius. The vortex circulation remains unchanged, indicating a diffusion mechanism that spreads concentrated vorticity over a larger core. At 12° incidence, the 9% gyroid insert reduced peak tangential velocity by a factor of 3.2 alongside a sixfold increase in vortex core radius. Consequently, the minimum pressure coefficient at the vortex center increased from $-1.4$ to $-0.1$, reducing significantly the risk of cavitation. Flow visualization confirmed complete suppression of tip vortex cavitation across all tested conditions. The gyroid effectiveness was verified for both tripped and natural boundary layer transitions. Critically, hydrodynamic performance remained essentially unaffected for gyroid inserts spanning up to 5%, with lift and drag coefficients maintained within experimental uncertainty. Tests with blocked permeability demonstrate that the observed effects stem from structural permeability rather than surface roughness. These findings establish gyroid inserts as a promising passive flow control for cavitation mitigation in marine propellers, hydrofoils, and turbomachinery, as well as noise control in aircraft wings and wind turbines, while preserving operational efficiency.

## Introduction

Tip vortices, generated at the tips of lifting surfaces such as wings, hydrofoils, and propeller blades, represent a significant source of induced drag, noise, and cavitation risk. These vortices arise from the pressure imbalance at the foil tip, causing the flow to roll up into a stable, concentrated core. This swirling flow induces downwash, which alters the local angle of attack and reduces lift, generating what is known as induced drag. For a modern commercial aircraft, this can account for up to 20% (Kroo 2005; Ma and Elham 2024) of the total drag during cruise, and the persistent wakes left by these vortices dictate the safe separation time for aircraft take-off and landing.

In hydraulic machinery (Arndt 2002; Arndt et al. 2015), particularly in axial turbines (Roussopoulos and Monkewitz 2000; Luo et al. 2016), the low pressure within the tip leakage vortices may cause cavitation, leading to noise, vibration, and material erosion. Tip vortices also play a critical role in other technologies: Their interaction with helicopter rotor blades (Yu 2000) can cause severe vibrations, while coupling with Kármán vortex shedding in wind turbines (Horcas et al. 2020) amplifies noise and structural loads. Consequently, mitigating tip vortices has been a long-standing goal in fluid dynamics. A multitude of strategies reviewed in detail by Platzer already in (1979) remain actively studied today. The most widely adopted technique is the winglet, now deployed on virtually all commercial aircraft. Originally proposed by Lanchester and modernized by Whitcomb in (1976), winglets improve lift-to-drag ratio by approximately 4 to 6% (Whitcomb 1976; NASA Spinoff 2015), saving hundreds of thousands of liters of fuel per aircraft annually. Other passive strategies include wing planform modification (Platzer and Souders 1979), an evolutionary adaptation also observed across bird species with different migration patterns (Lockwood et al. 1998). Inspired by Prandtl’s (1933), Bowers et al. (2016) proposed a design with upwash at the wingtips to eliminate the abrupt transition from downwash to upwash, thereby significantly reducing induced drag and even generating induced thrust for yaw control.

In axial hydroturbines, anti-cavitation lips, consisting of simple plates attached to the blade tips, are commonly used as rudimentary winglets to mitigate tip vortex cavitation (TVC). However, such a device is not always efficient (Roussopoulos and Monkewitz 2000; Amini, et al. 2019a, b). Other methods, including true winglets (Amini et al. 2019a, b; Gao et al. 2019), overhanging grooves (Dreyer 2015; Cheng et al. 2020), added roughness (Asnaghi et al. 2020), or flexible thread (Amini et al. 2019a, b) attached to the wing tip, have shown promising ability to thicken the vortex core and thereby increase the core pressure. Despite their laboratory success, these techniques remain confined to experimental demonstrations and have not found practical industrial applications.

Porous tips, as a means to mitigate tip vortices, have attracted attention in both aerodynamic and hydraulic applications for over five decades (Thompson 1973; Platzer and Souders 1979; Smith 1980; Sharma et al. 1990; Aktas et al. 2020; Aldheeb et al. 2020; Bi et al. 2024). Smith was the first to demonstrate in (1980) that porosity shifts the lift distribution inboard and reduces vortex tangential velocities. Subsequent research on porous treatments for tip vortex mitigation, using distributed holes or open-cell foam, has focused primarily on underwater radiated noise from ship propellers (Sharma et al. 1990; Aktas et al. 2020; Aldheeb et al. 2020), consistently confirming these findings.

We have recently demonstrated (Berger and Farhat 2025) that a gyroid-based porous insert attached to a hydrofoil’s trailing edge is remarkably effective in eliminating vortex shedding and the associated resonance and lock-in phenomena. In the present work, we propose to extend the application of gyroid inserts to the control of tip vortex flows. To this end, we designed gyroid-based porous inserts to replace the plain tip of an elliptical hydrofoil and measure their effect through cavitation inception and velocity field comparison.

**Fig. 1:**
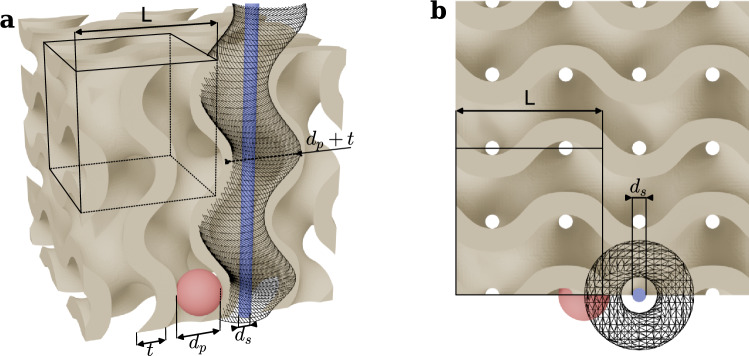
Gyroid sheet-based geometry with *L* = 2 mm and *t* = 0.25 mm. Red sphere showing the pore diameter *d*_*p*_ = 0.75 mm, blue cylinder showing see-through pores with *d*_*s*_ = 0.2 mm and helicoidal wireframe show an approximation of the gyroidal pore geometry with a sweep amplitude of *A*_*p* _= 0.5 mm. **a** Perspective view. **b** top view with orthographic projection

Gyroids belong to the family of triply periodic minimal surfaces, discovered by Schoen (1970), that gained popularity with the rise of additive manufacturing due to their excellent isotropic structural properties and minimal material usage. They are now extensively used across diverse applications: Their interwoven dual-pore network structure enables efficient heat transfer and chemical reactions for heat exchangers (Yeranee and Rao 2022) and battery engineering (Lee et al. 2019; Du 2025), while their high porosity-to-strength ratio makes them ideal for bone implants (Yan et al. 2015). The pores can be approximated by two networks of left- and right-handed helicoids, providing a high tortuosity compared to the other porous flow control in the literature. To obtain a volumetric object, the surface is thickened to create a sheet-based gyroid, as presented in Fig. 1(a). The pore size *d*_*p*_ is determined by the gyroid unit cell $L$ and the surface thickness $t$, while the helicoid sweep amplitude is defined only by the gyroid unit cell size. Therefore, using a shallow surface thickness yields see-through pores with a diameter ${d}_{s}$ with high permeability as shown in Fig. 1(b), while still providing higher tortuosity than conventional porous media. Increasing the thickness further eliminates these through pores and substantially reduces permeability while further increasing tortuosity. Permeability is a foundational mechanism in this tip vortex mitigation; however, reducing gyroids to a single idealized Darcy number for such complex macroscopic channel structure has proven to be highly challenging. A more general characterization framework, which accounts for anisotropic multidirectional permeability, Forchheimer effects, entrance/exit effects, and morphological features such as tortuosity, is currently lacking in the state of the art.

## Methods

### Test facility and instrumentation

The tests were carried out in the high-speed cavitation tunnel at EPFL, which features a 150 mm×150 mm×750 mm test section. The facility, described in detail in (Dupont 1991; Ausoni 2009), supports a maximum freestream velocity of 50 m/s with fluctuations below 1% and a maximum static pressure of 16 bar.

The cavitation number $\sigma$ and the Reynolds number Re are defined as follows:

$$ \sigma = \frac{{p_{\infty } - p_{v} \left( T \right)}}{{\frac{1}{2}\rho V_{\infty }^{2} }},{ }\,{\mathrm{Re}} = \frac{{\rho V_{\infty } C_{0} }}{\mu } $$

where ${C}_{0}$ is the chord length at the hydrofoil root, ${p}_{\infty }$ and ${V}_{\infty }$ are, respectively, the static pressure and freestream velocity, measured at the test section inlet, $\rho$ and $\upmu$ are, respectively, the water density and the water dynamic viscosity, and ${p}_{v}\left(T\right)$ is the saturation vapor pressure of water at temperature $T$. All measurements presented in this paper were performed with inlet velocity ${V}_{\infty }=15 \mathrm{m}/\mathrm{s}$, resulting in $Re\approx 9\times {10}^{5}$.

The lift and drag forces are measured using a 5-component load cell described in (Dupont 1991) with a maximum measurable lift force of 10^4^ N and a precision of 1.5 N and 0.5 N for the lift and drag, respectively. The mean value of the lift and drag coefficients was measured during 2 s, sampled at 1000 Hz in a non-cavitating flow. The lift and drag coefficients are defined as follows:

$$ C_{L} = \frac{{{\mathrm{Lift}}}}{{0.5{\uprho }V_{\infty }^{2} A_{ref} }},{ }C_{D} = \frac{{{\mathrm{Drag}}}}{{0.5{\uprho }V_{\infty }^{2} A_{ref} }} $$

where ${V}_{\infty }$ is the freestream velocity, $\rho$ is the water density, and ${A}_{ref}$ is the planform area of the hydrofoil.

Flow visualization is used for assessing tip vortex cavitation mitigation using still photography with a 30-µs duration flash lamp.

### Hydrofoils and gyroid-based insert

The baseline hydrofoil is an elliptical planform with NACA 16–020 cross section defined by Eq. (1) where ${z}_{b}$ represents the thickness distribution, ${x}_{b}$ the chordwise coordinate, and $C(y)$ the chord length as function of the spanwise position, forming the elliptical shape. The root chord measures ${C}_{0}=60 \mathrm{m}\mathrm{m}$, and the initial span $S=90 \mathrm{m}\mathrm{m}$ is trimmed to 90%, leaving $9 \mathrm{m}\mathrm{m}$ to be replaced with our gyroid tip insert. The reference area for the determination of lift and drag coefficients is ${A}_{ref}=42.57\times {10}^{-4} {\mathrm{m}}^{2}$.

1 $$ \begin{gathered} \left\{ {\begin{array}{*{20}c} {\frac{{z_{b} }}{C\left( y \right)} = a_{0} \left( {\frac{{x_{b} }}{C\left( y \right)}} \right)^{\frac{1}{2}} + a_{1} \left( {\frac{{x_{b} }}{C\left( y \right)}} \right) + a_{2} \left( {\frac{{x_{b} }}{C\left( y \right)}} \right)^{2} + a_{3} \left( {\frac{{x_{b} }}{C\left( y \right)}} \right)^{3} } \\ {\frac{{z_{b} }}{C\left( y \right)} = b_{0} + b_{1} \left( {1 - \frac{{x_{b} }}{C\left( y \right)}} \right) + b_{2} \left( {1 - \frac{{x_{b} }}{C\left( y \right)}} \right)^{2} + b_{3} \left( {1 - \frac{{x_{b} }}{C\left( y \right)}} \right)^{3} } \\ \end{array} } \right. \begin{array}{*{20}c} {0 \le \frac{{x_{b} }}{C\left( y \right)} \le 0.5} \\ {0.5 \le \frac{{x_{b} }}{C\left( y \right)} \le 1} \\ \end{array} \hfill \\ \begin{array}{*{20}l} {a_{0} = + 0.197933} \hfill & {a_{1} = - 0.047850} \hfill & {a_{2} = - 0.00820} \hfill & {a_{3} = - 0.111880} \hfill \\ {b_{0} = + 0.002} \hfill & {b_{1} = + 0.465} \hfill & {b_{2} = - 0.684} \hfill & {b_{3} = + 0.292} \hfill \\ \end{array} \hfill \\ \end{gathered} $$

It is well established that the boundary layer state has a significant influence on tip vortex formation and development. Since our hydrofoils have hydraulically smooth surfaces at all tested velocities, the natural boundary layer would remain laminar over a substantial portion of the chord. To also examine the effect of a turbulent boundary layer, we apply a leading-edge trip in the form of a distributed roughness following Dreyer (2015). The hydrofoil equipped with this trip will be referred to as the rough hydrofoil.

**Fig. 2:**
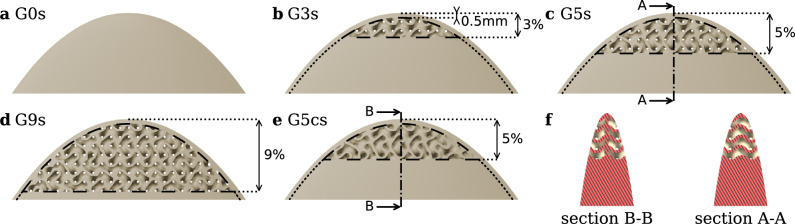
Detailed view of the gyroid inserts. **a**–**d** 3D models of the 0%(G0s), 3%(G3s), 5%(G5s), and 9%(G9s) inserts. **e** Cross section of the 5% (G5s) and 5% closed (G5Cs) inserts showing the blocked permeability

**Fig. 3a,:**
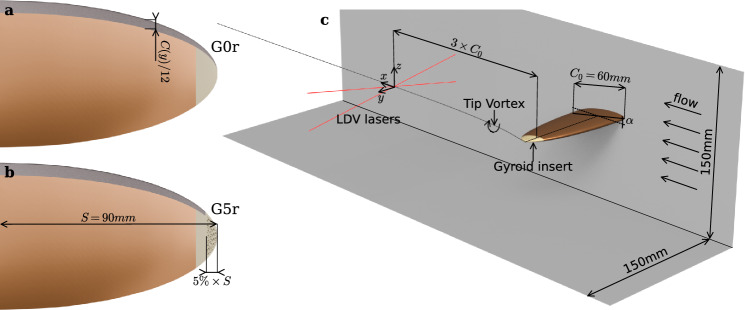
**b** Top view of the hydrofoil equipped with G0r and G5r inserts with the distributed roughness element at the leading edge; **c** isometric view of the experimental setup, NACA 16-020 hydrofoil with gyroid insert and LDV measurement

The insert consists of a 3D-printed porous gyroid-shaped tip extension. The gyroid is approximated using a marching cube algorithm with the following level-set equation (Berger and Farhat 2025):

2 $$ \varphi \left( {x,y,z} \right) = \sin \left( {\lambda x} \right)\cos \left( {\lambda y} \right) + \sin \left( {\lambda y} \right)\cos \left( {\lambda z} \right) + \sin \left( {\lambda z} \right)\cos \left( {\lambda x} \right) = 0 $$

where $\lambda = 2 \pi / L$ and $L$ corresponds to the cubic unit cell (spatial period) in each direction. The final extension is generated using a unit cell $L=2 \mathrm{m}\mathrm{m}$. The surface is then thickened with a wall thickness of $t=0.25 \mathrm{m}\mathrm{m}$ using (Blender and Farhat 2025) to obtain a gyroid sheet-based volume. As illustrated in Fig. 1, this design yields networks of helicoidal pores whose diameter matches the tip vortex diameter with ${d}_{p}=L/2-t=0.75 \mathrm{m}\mathrm{m}$ and a sweep amplitude of ${A}_{p}=L/4=0.5 \mathrm{m}\mathrm{m}$, leading to very small see-through pore of ${d}_{s}\approx 0.2 \mathrm{m}\mathrm{m}$ and a very tortuous geometry. The resulting insert is placed at the hydrofoil tip and trimmed with respect to the original hydrofoil geometry. As shown in Fig. 2(a), to maintain consistent tip geometry regardless of gyroid placement, a $0.5-\mathrm{m}\mathrm{m}$ solid band is preserved around the tip perimeter. The insert is then 3D printed with stereolithography Formlabs Form 3. The gyroid-shaped insert is finally glued carefully to the truncated tip. This results in a highly permeable geometry, and thus, any trapped air bubbles are easily evacuated as soon as flow is initiated. As illustrated in Fig. 2(b–e), four inserts with increasing spanwise gyroid portions were tested: G0s (0%, non-permeable insert—baseline), G3s (3% span), G5s (5% span), and G9s (9% span). The s suffix denotes the smooth hydrofoil configuration, indicating a natural transition to turbulence. Additionally, a fifth insert G5Cs was also manufactured, similar to G5s but closed with a thin solid partition added in the insert mid-plane blocking flow through the insert as shown in Fig. 2(f). When using a tripped boundary layer (rough hydrofoil), the distributed roughness was also applied on the insert up to the gyroid element as shown in Fig. 3(a, b). The tripped insert will be referred to as G0r, G3r, G5r, G9r, G5Cr.

### Vortex model

To represent the vortex flow, we use the Vatistas vortex model (1991) to relate the tangential velocity to the radial position $r$, using the vortex strength $\Gamma$, its core radius ${r}_{c}$, and a shape parameter $n$ as follows:

3 $$ V_{\theta } \left( r \right) = \frac{\Gamma }{2\pi } \cdot \frac{r}{{\left( {r_{c}^{2n} + r^{2n} } \right)^{1/n} }} $$

**Fig. 4:**
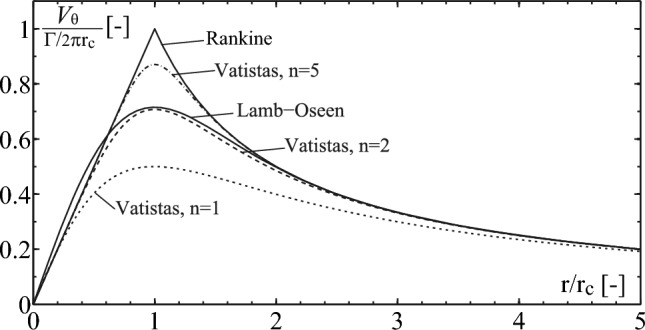
Illustration of the tangential velocity given by different vortex models with the same circulation $\Gamma$ (Dreyer 2015)

The shape parameter $n$ provides more flexibility than the classic Lamb–Oseen model as illustrated in Fig. 4. The minimum pressure coefficient at the vortex center, ${C}_{{p}_{min}}$, can be obtained by integrating the radial equilibrium equation. We can derive the following relationships:

4 $$ \begin{gathered} V_{{\theta_{\max } }} = V_{\theta } \left( {r = r_{c} } \right) = \frac{\Gamma }{{2\pi \cdot r_{c} \cdot 2^{1/n} }} \hfill \\ p\left( 0 \right) - p_{\infty } = \rho \mathop \smallint \limits_{0}^{\infty } \frac{{V_{\theta }^{2} }}{r}\,{\mathrm{d}}r = - \rho \left( {\frac{\Gamma }{2\pi }} \right)^{2} \cdot \frac{1}{{nr_{c}^{2} }}\mathop \smallint \limits_{0}^{\infty } \frac{{s^{2/n - 1} }}{{\left( {1 + s^{2} } \right)^{2/n} }}\,{\mathrm{d}}s \hfill \\ C_{{p_{\min } }} = \frac{{p|_{r = 0} - p_{\infty } }}{{0.5\rho V_{\infty }^{2} }} = - \frac{{\beta \left( {\frac{1}{n},\frac{1}{n}} \right)}}{{4\pi^{2} n}} \cdot \left( {\frac{\Gamma }{{V_{\infty } r_{c} }}} \right)^{2} \hfill \\ \end{gathered} $$

where $\beta$ denotes the beta function (Euler integral of the first kind).

### LDV velocity measurement and fitting routine

Flow velocity measurements were taken using Dantec Flow Explorer single-point 2-component Laser Doppler Velocimeter (LDV), which has an accuracy of 0.11% (coverage factor 2), with 10-µm hollow glass spheres as seeding particles. The measurement origin was set at $3\times {C}_{0}$ downstream of the hydrofoil tip, as shown in Fig. 3, and was precisely positioned on the mean vortex center. Following the methodology described by Dreyer (2015), the vortex center was identified by recording the *z*-velocity component along two axes aligned with the *z*-axis and *y*-axis, respectively. The measurement origin is finally set by locating the zero crossing of the y-axis scan and the extremum of the *z-*axis scan. The tangential velocity of the vortex was measured using the *z*-velocity component while moving the optical head along the *y*-axis using a laser wavelength of $660 \mathrm{n}\mathrm{m}$ resulting in a water measurement volume of ${\delta}_{x}\times {\delta}_{y}\times {\delta}_{z}=0.12\times 1.20\times 0.12 \mathrm{m}\mathrm{m}$. At each measurement location, 5000 particle velocity samples were collected, and the complete statistical distribution is used to estimate the velocity profile.

When measuring the vortex velocity profile, we need to take into account two sources of positional uncertainties. First, vortices exhibit random oscillations of their centerline, known as wandering. These oscillations can be modeled as 2D Gaussian probability distribution with a spherical variance ${\upsigma}_{w}$ as proposed by Devenport et al. (1996). Second, the precise location of the measured particle inside the measurement volume is inherently uncertain. With two Gaussian laser beams, the probability distribution of the particle’s position can also be modeled by a 2D Gaussian probability function with an elliptical variance ${\sigma}_{y},{\sigma}_{z}$. The combined effect of these uncertainties results in the convolution of the respective Gaussian distributions with the true velocity field (Dreyer 2015), as shown in Eq. (5).

5 $$ \begin{gathered} V_{\theta }^{c} = {\mathrm{PDF}}_{v} *{\mathrm{PDF}}_{w} *V_{\theta } \hfill \\ {\mathrm{PDF}}_{v} *{\mathrm{PDF}}_{w} \left( {y_{v} ,z_{v} } \right) = \frac{1}{{2\pi \sqrt {\sigma_{w}^{2} + \sigma_{vy}^{2} } \sqrt {\sigma_{w}^{2} + \sigma_{vz}^{2} } }}\exp \left( { - \frac{1}{2}\left( {\frac{{y_{v}^{2} }}{{\sigma_{{w^{2} }} + \sigma_{{vy^{2} }} }} + \frac{{z_{v}^{2} }}{{\sigma_{{w^{2} }} + \sigma_{{vz^{2} }} }}} \right)} \right). \hfill \\ \end{gathered} $$

where ${V}_{\uptheta }^{c}$ is the convoluted velocity, ${V}_{\uptheta }$ is the true velocity, $\mathrm{P}\mathrm{D}\mathrm{F}$ is a bivariate normal probability density function, $*$ is to the convolution operation, $y \mathrm{a}\mathrm{n}\mathrm{d} z$ are coordinates, $\upsigma$ is the variance, the subscript $v$ corresponds to the measurement particle position inside the LDV measurement volume, and the subscript $w$ corresponds to the wandering.

**Fig. 5a:**
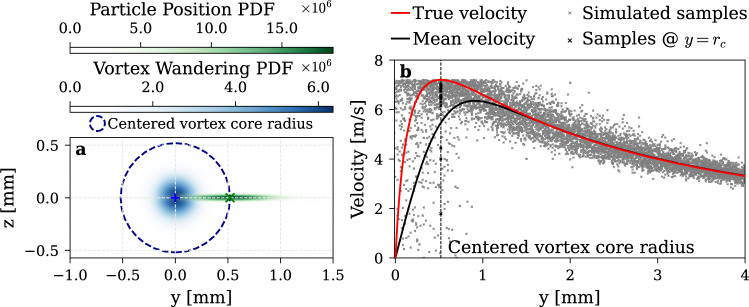
Wandering and measurement particle position PDFs and **b** true vs. mean velocity profiles, along with simulated samples illustrating the smoothing effect from positional uncertainties for the G0 insert

Both phenomena generate a smoothing effect on the mean measurement leading to a more diffused measurement (Devenport et al. 1996; Dreyer 2015) with an underestimation of the maximum tangential velocity ${V}_{{\theta}_{max}}$ and an overestimation of the vortex core radius ${r}_{c}$ as illustrated in Fig. 5. Notably, when the measurement is positioned at the vortex core radius (${y}_{LDV}={r}_{c}$), the system is most likely to capture the maximum tangential velocity. However, it may also include lower velocities either from within or outside the vortex core, or from instances where the instantaneous vortex position and the measurement point are misaligned along the *z*-axis leading to a lower projected velocity being recorded.

One limitation of the wandering model is the assumption of perfect 2D translation of the vortex core. When the measurement point is close to the vortex core radius, within a wandering amplitude of the vortex center, there exists a finite probability of capturing the maximum tangential velocity. However, velocities exceeding this maximum cannot be recorded. This nonlinearity introduces a hard ceiling in the tangential velocity distribution, visible in Fig. 5. A more physically realistic scenario would account for the fact that, as the vortex wanders, its axis may no longer remain aligned with the streamwise direction, requiring the vortex to stretch and bend. These three-dimensional deformations would produce a smoother Probability Density Function in the measured velocity distributions. Nevertheless, despite its inability to fully reproduce the realistic shape of the PDF, the 2D wandering model remains sufficient to accurately recover the true mean velocity profile and to better capture the pressure deficit at the vortex core, which is essential for the reliable prediction of Tip Vortex Cavitation inception.

In this work, we adopt the standard forward deconvolution approach. This method relies on a theoretical instantaneous vortex model which is convoluted, and its parameters are iteratively adjusted to achieve the best fit to the mean experimental measurement. This method provides a stable reconstruction of the deconvoluted vortex with limited noise amplification, and when an appropriate vortex model is used, directly yields the relevant physical parameters. Alternative deconvolution methods have been proposed (Bailey and Tavoularis 2008; Dreyer 2015), but they all require multi-point measurements. 

To ensure a physically relevant reconstructed instantaneous vortex, certain fitting parameters must be constrained. In most approaches, the wandering amplitude ${\upsigma}_{w}$ is constrained using independent measurements such as PIV (Dreyer 2015) or estimated from the standard deviation of the LDV measurements at the vortex center (Devenport et al. 1996), under the assumption of a laminar vortex core, a condition that cannot be guaranteed in the present experiment. In this work, the forward deconvolution is instead constrained using estimates of the maximum tangential velocity ${V}_{{\theta}_{max}}$ and the vortex core radius ${r}_{c}$, derived directly from the experimental data. As illustrated in Fig. 5, the maximum tangential velocity is unaffected by vortex wandering and cannot be overestimated by LDV measurement. The peak tangential velocity is measured when the LDV measurement volume is located at a radial distance exactly equal to ${r}_{c}$ from the instantaneous vortex center, aligned with the measurement axis. The likelihood of capturing this peak velocity is therefore maximized when the LDV volume is centered at $\left(y,z\right)=\left({r}_{c},0\right)$. Consequently, ${r}_{c}$ can be retrieved as the radial location where the peak tangential velocity is most frequently observed. By taking an upper bound of the cumulative Probability Density Function (PDF) of the LDV measurements, data points that underestimate the maximum tangential velocity ${V}_{{\theta}_{max}}$ and overestimate the vortex core radius ${r}_{c}$ are effectively filtered out. Upper bounds of 90%, 95%, and 99% were tested, yielding very similar results; the 90% threshold was retained as it proved less susceptible to measurement noise.

The means and standard deviations of the LDV measurements are referred to as $\overline{{\mathrm{v} }_{\uptheta }}=\mathrm{m}\mathrm{e}\mathrm{a}\mathrm{n}\left({\mathrm{v}}_{\uptheta }\right)$ and $v_{\theta }{\prime} = {\mathrm{stddev}}\left( {v_{\theta } } \right)$, respectively. When referring to a measurement obtained with a specific insert, the insert name is added as a superscript. The maximum tangential velocity is defined as ${\mathrm{v}}_{{\theta}_{max}, \mu }=\mathrm{m}\mathrm{a}\mathrm{x}\left(\overline{{\mathrm{v} }_{\uptheta }}\right)$ for the mean vortex and ${{\mathrm{v}}_{{\theta}_{max}, {D}_{9}}=\mathrm{m}\mathrm{a}\mathrm{x}(D}_{9}\left[{v}_{\uptheta }\left(r\right)\right]$ for the decile vortex. The vortex core radius, defined as the radial location of the peak tangential velocity, is defined as ${r}_{c,\mu }=\mathrm{a}\mathrm{r}\mathrm{g}\mathrm{m}\mathrm{a}\mathrm{x}\left(\overline{{\mathrm{v} }_{\uptheta }}\left(r\right)\right)$ for the mean measurement and ${r}_{c,{D}_{9}}=\mathrm{a}\mathrm{r}\mathrm{g}\mathrm{m}\mathrm{a}\mathrm{x}({{D}_{9}[v}_{\uptheta }(r)])$ for the decile vortex.

The overall procedure consists of (1) extracting ${V}_{{\theta}_{max}}$ and ${r}_{c}$ from the 9th decile of the LDV PDF, i.e., ${\mathrm{v}}_{{\theta}_{max}, {D}_{9}} and {r}_{c,{D}_{9}}$; (2) using these values as constraints in the fitting equations (Eq. (3) and (5)), and (2) adjusting the shape parameter $n$ and the wandering amplitude ${\upsigma}_{w}$ such that the convoluted vortex matches the mean experimental measurement.

## Results and discussion

### Effect of a gyroid-insert on tip vortex cavitation (TVC)

**Fig. 6:**
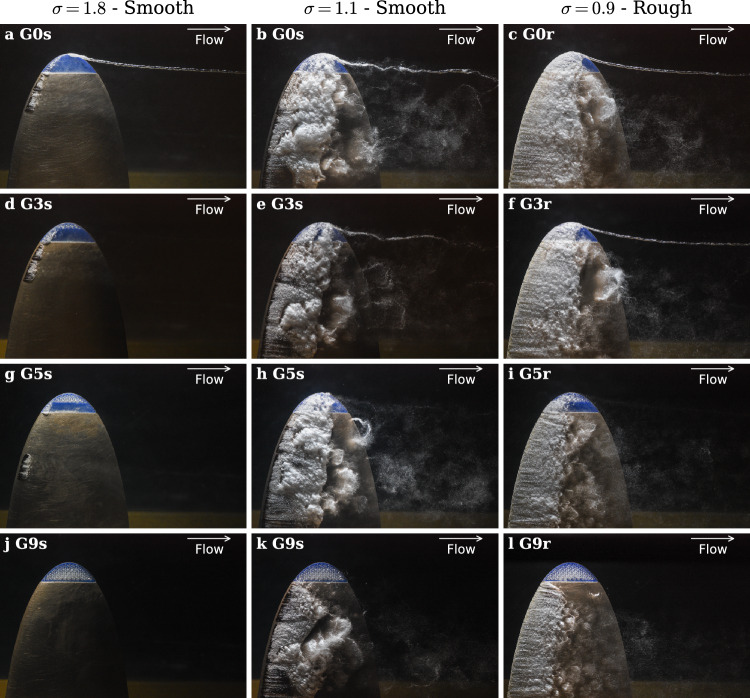
Top views of cavitation development for natural (smooth leading edge) and tripped (rough leading edge) boundary layers with increasing gyroid portion (0%, 3%, 5%,9%). $\mathrm{R}\mathrm{e}=9\times {10}^{5}$ and $\alpha =12^\circ$

We observed the cavitation inception and development for the gyroid inserts G3S, G5S, and G9S and compared them with the baseline G0S (plain insert). The results are shown in Fig. 6 for an upstream velocity of $12 \mathrm{m}/\mathrm{s}$ and an incidence angle of 12°. For $\sigma = 1.8$, TVC is well developed for G0S, accompanied by incipient leading-edge cavitation. All gyroid inserts completely suppress TVC. Interestingly, the leading-edge cavitation is also reduced with the G5S insert and fully suppressed with G9S. For $\sigma = 1.1$, the baseline hydrofoil exhibits fully developed TVC and cloud cavitation. In this regime, TVC is notably suppressed by both the G5S and G9S inserts. For $\sigma = 0.9$ (roughened leading edge), the increased boundary-layer turbulence produces the characteristic foamy appearance of the leading-edge cavitation. Again, the G5r and G9r inserts completely eliminate TVC. At low cavitation numbers (Fig. 6e, f), the leading-edge cavitation cavity extends onto the gyroid insert, which we believe restricts flow through the insert’s permeable geometry, producing a saturation effect that undermines TVC mitigation. With a larger gyroid portion (Fig. 6k, i), the insert remains cavitation-free and limits the spanwise extent of the leading-edge cavitation cavity, successfully eliminating TVC. We further observe that the extent of leading-edge cavitation decreases systematically as the gyroid span increases. These results are confirmed for other flow conditions.

### Measurements of the velocity field

**Fig. 7:**
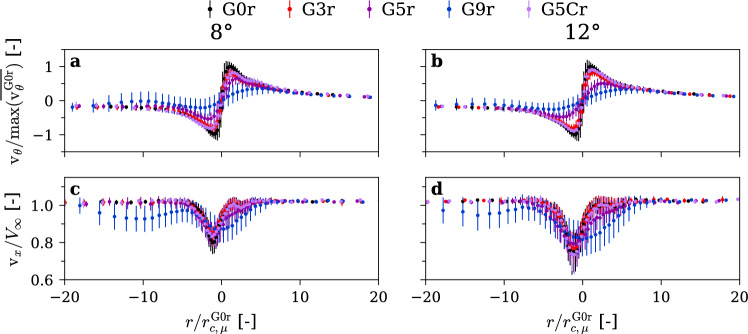
Mean and standard deviation of tangential (**a**, **b**) and streamwise (**c**, **d**) velocity profiles along the radial axis for increasing gyroid tip portion (0%, 3%, 5%, 9%) at incidence angles of 8°(**a**, **c**) and 12°(**b**, **d**). $\mathrm{R}\mathrm{e}=9\times {10}^{5}$, rough hydrofoil

We used LDV to measure the streamwise and tangential velocity components across the vortex center at a downstream location of $3\times {C}_{0}$ from the hydrofoil tip. The means $\overline{{\mathrm{v} }_{\uptheta }}$ and standard deviations $v^{\prime}_{\theta }$ of the velocity components are presented in Fig. 7 for rough hydrofoils. A clear trend emerges, increasing the gyroid portion of the hydrofoil tip decreases the maximum tangential velocity $\mathrm{m}\mathrm{a}\mathrm{x}(\overline{{\mathrm{v} }_{\uptheta }})$ while increasing the vortex core radius, ${r}_{c}$. For instance, with the G9r insert at an incidence angle of 12°, $\mathrm{m}\mathrm{a}\mathrm{x}(\overline{{\mathrm{v} }_{\uptheta }})$ is reduced by a factor of 3.2, alongside a sixfold increase in ${r}_{c}$. Outside the vortex core, however, the velocity profiles remain nearly identical, indicating that the overall vortex circulation is essentially unchanged. This suggests that the gyroid tip does not modify the vortex strength, but instead diffuses the concentrated vorticity, thereby enlarging the vortex core and reducing the peak tangential velocity. The same behavior was observed at lower incidence angles: Increasing the gyroid portion consistently reduces $\mathrm{m}\mathrm{a}\mathrm{x}(\overline{{\mathrm{v} }_{\uptheta }})$ and increases ${r}_{c}$.

The axial velocity profiles in Fig. 7 exhibit a wake-like distribution under all conditions, which is consistent with the observations of Dreyer (2015) for rough hydrofoils. As the gyroid tip portion increases, the velocity deficit becomes distributed over a larger vortex core, resulting in a reduced minimum axial velocity.

**Fig. 8:**
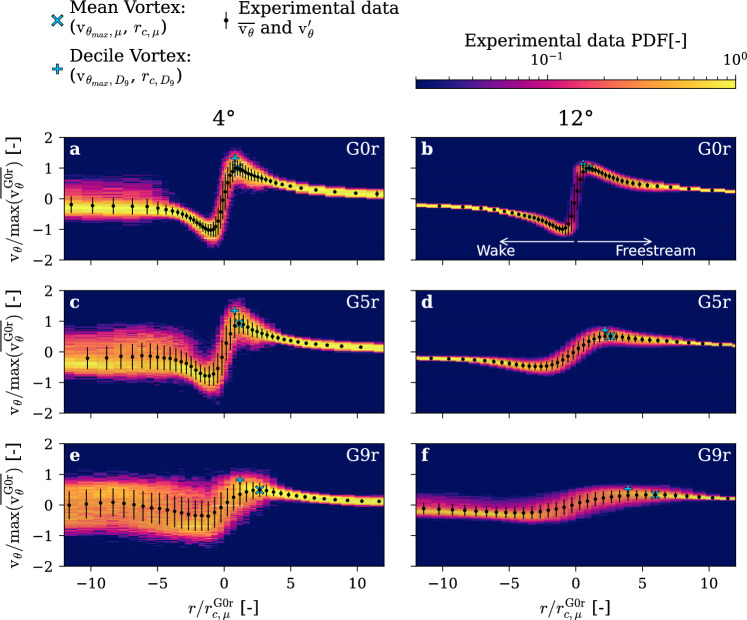
Subset of LDV measurements with increasing gyroid tip portion 0% (**a**, **b**), 5% (**c**, **d**), 9% (**e**, **f**) at incidence angles of 4° (**a**, **c**, **e**), and 12° (**b**, **d**, **f**). Probability Density Function of the tangential velocity along the radial position, along with the mean and standard deviation, and the peak tangential velocity and its radial location for the mean and the 9th decile vortex. $\mathrm{R}\mathrm{e}=9\times {10}^{5}$, rough hydrofoil

Figure 8 shows a representative subset of LDV measurements, providing the measured Probability Density Function (PDF) and the retrieved mean and standard deviation. As detailed in the Methods section, the LDV measurement can underestimate the tangential velocity due to positional uncertainties, making a two-dimensional Probability Density Function an appropriate way to represent the data. As clearly visible in all cases, the mean vortex shows the resulting smoothing effect. Nevertheless, the overall trends are well captured by the mean vortex, and the behaviors identified in Fig. 7 remain consistent.

It is unlikely that the positional uncertainties lead to an overestimation of the maximum tangential velocity, which typically occurs at the vortex core radius. To better estimate the maximum tangential velocity and the vortex core radius, we consider the upper decile of the cumulative tangential velocity distribution, termed here as the “decile vortex.” This approach mitigates the smoothing effect of vortex wandering. Figure 8 illustrates both the maximum tangential velocity ${V}_{{\uptheta}_{max}}$ and its radial position ${r}_{c}$ for the mean vortex and for the decile vortex on the freestream side. As observed previously in Fig. 7, increasing the gyroid tip portion leads to a significant increase in ${r}_{c}$ and a decrease in ${V}_{{\theta}_{max}}$. Indeed, the decile vortex offers a more accurate estimation of these parameters, unaffected by the uncertainty smoothing. More interestingly, the smoothing effect appears to intensify as the gyroid portion increases, suggesting an increase of the vortex wandering.

Moreover, at an incidence angle of 4°, the wake turbulence becomes visible as it interacts with the tip vortex due to reduced downwash. However, with the non-permeable tip (G0r), the vortex remains unaffected by this turbulence, resulting in a narrow vortex profile. With an increasing gyroid portion, at both 4° and 12° of incidence, the relative amount of turbulence at the vortex core radius increases. At 4° of incidence, the wake turbulence and the tip vortex mix and produce a very wide distribution of velocities, spanning from the wake up to the tip vortex.

**Fig. 9:**
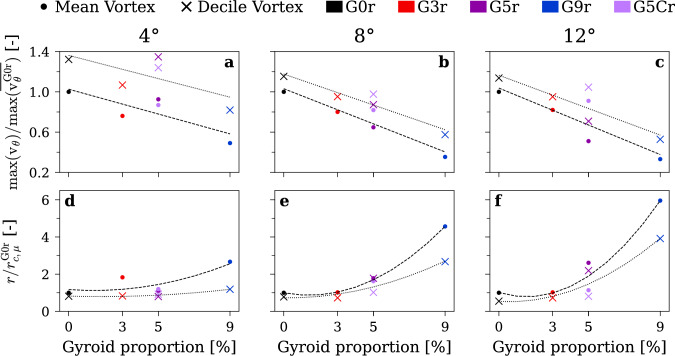
Parameters extracted from the measured PDF for increasing gyroid tip portion. Maximum tangential velocity (**a**, **b**, **c**) and vortex core radius (**d**, **e**, **f**) at incidence angles of 4° (**a**, **d**), 8° (**b**, **e**), and 12° (**c**, **f**). $\mathrm{R}\mathrm{e}=9\times {10}^{5}$, rough hydrofoil. Colors redundantly indicate gyroid tip spanwise portion for clarity

Figure 9 presents the maximum tangential velocity and the vortex core size, extracted from the measurement in Fig. 8. Consistent with the findings in Fig. 7, increasing the gyroid tip portion decreases the maximum tangential velocity and increases the vortex core size. At an incidence angle of 8° or 12°, the maximum tangential velocity decreases nearly linearly, and the vortex core radius increases approximately quadratically, with increasing gyroid tip portion. At 12° of incidence, increasing the gyroid portion to 5% and 9% results in a reduction of tangential velocity by factors of 2 and 3, respectively. Although the trend is less pronounced at the lower incidence angle of 4°, the G9r insert still produces a notable decrease in tangential velocity and a corresponding increase in vortex core size.

### Vortex modeling

**Fig. 10:**
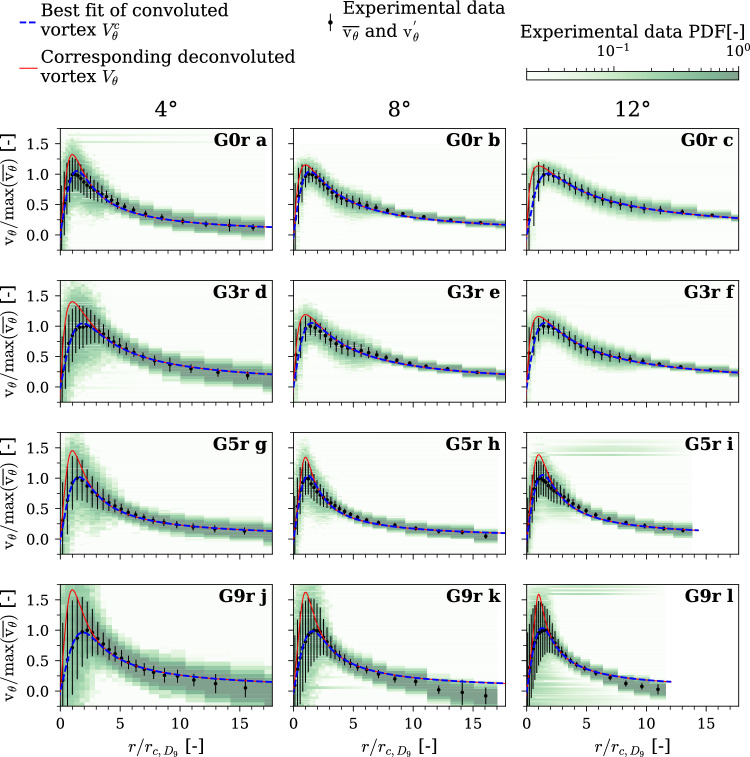
Fitting procedure results with the instantaneous vortex profile resulting from ${V}_{{\theta}_{max}}$ and ${r}_{c}$ of the experimental decile vortex, the fitted mean vortex profile computed with the positional uncertainties, along with Probability Density Function, mean, and standard deviations of the LDV measurement

Figure 10 summarizes the experimental data $\overline{{v }_{\theta }}$ *and* $v^{\prime}_{\theta }$ along with the best fit of the convoluted model ${V}_{\theta }^{c}$ and its corresponding deconvolution ${V}_{\theta }$ (see Eq. (5)); the experimental PDF is shown in the background for reference. For each experimental condition, two profiles are shown: The blue curve shows the convoluted vortex representing the mean vortex, accounting for the positional uncertainties of the LDV measurement and fitted wandering amplitude ${\upsigma}_{w}$. The red curve shows the corresponding deconvoluted vortex model, representing the experimental decile vortex passing through (${r}_{c}$, ${V}_{{\theta}_{max}}$). Across all conditions, the fitted mean vortex aligns closely with the mean experimental measurements near the core ($x/{r}_{c}<10$). Once again, the smoothing effect of positional uncertainties is evident: The mean vortex exhibits a substantially lower peak tangential velocity than the instantaneous vortex, and this attenuation is stronger when the experimental standard deviation is larger.

**Fig. 11:**
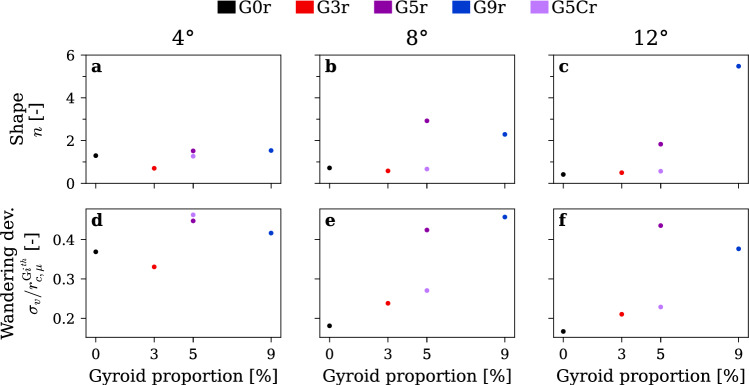
Resulting fitted parameters for increasing gyroid tip portion. The Vatistas shape parameters (**a**–**c**) and the vortex wandering normalized by the vortex core radius (**d**–**f**) at incidence angles of 4° (**a**, **d**), 8° (**b**, **e**), and 12° (**c**, **f**). $\mathrm{R}\mathrm{e}=9\times {10}^{5}$, rough hydrofoil. Colors redundantly indicate gyroid tip spanwise portion for clarity

Figure 11 presents the parameters obtained from the fitting procedure. The Vatistas shape parameter, shown in Fig. 11(a–c), increases as the gyroid tip portion is increased, and this trend becomes more pronounced with higher incidence angles. Specifically, the shape parameter rises from $n=0.5$ to above $n=5$ for the G9r insert at 12° of incidence, indicating a significant transformation in vortex morphology. This highlights the suitability of the Vatistas vortex model, whereas simpler models such as the Lamb–Oseen would fail to accommodate this additional degree of freedom. As presented in Fig. 11(d–f), the relative wandering exhibits a marked step increase reaching $0.4{r}_{c}$ when increasing the gyroid portion to 5% and then remains approximately constant as the tip portion is increased to 9%. At low incidence of 4°, this step is less pronounced with the G0r insert already exhibiting a relative wandering of about $0.35{r}_{c}$. However, at higher incidence the step is more visible as the relative wandering for G0r and G3r inserts is around $0.2{\mathrm{r}}_{\mathrm{c}}$. Additionally, a 3% gyroid portion causes a slight increase in relative wandering at both 8° and 12° incidence, likely due to the increased surface roughness that introduces turbulence into the vortex core. We believe that the high tip permeability combined with the high tortuosity of the gyroid pattern drives the large and sharp increase of relative wandering when passing the 5% gyroid tip portion. This increase of vortex wandering, and consequently its smoothing effect, further reduces the mean tangential velocity of the vortex. These findings point to a critical threshold in gyroid tip design, beyond which pronounced changes in vortex dynamics can be achieved.

**Fig. 12:**
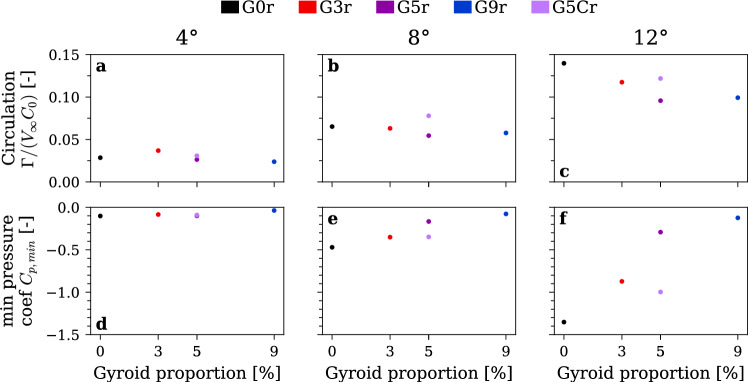
Parameters computed from the vortex model fit for increasing gyroid tip portion. Vortex strength (**a**–**c**) and minimum pressure coefficient in the vortex center (**d**–**f**) at incidence angles of 4° (**a**, **d**), 8° (**b**, **e**), and 12° (**c**, **f**). $\mathrm{R}\mathrm{e}=9\times {10}^{5}$, rough hydrofoil. Colors redundantly indicate gyroid tip spanwise portion for clarity

Figure 12 presents vortex properties derived from the vortex model. The vortex strength and the minimum pressure coefficient are computed from the fitted parameters using Eq. (4). As shown in Fig. 12(a, b), at incidence angles of 4° and 8°, the vortex strength remains relatively constant as the proportion of the gyroid tip increases. At higher incidence angle of 12° Fig. 12(c), a slight decrease in circulation is found while increasing the gyroid portion. We attribute this to the gyroid’s high tortuosity and permeability, which redistribute some of the vorticity generated at the tip into a broader and more diffuse wake, which is not fully captured by the localized LDV scan of the concentrated vorticity. Nevertheless, the vortex strength remains nearly unchanged, so the main reduction of tangential velocity can be attributed to the increase of the vortex core size. This means that the gyroid insert helps diffuse the vorticity generated at the tip and reduce the associated cavitation risk. As presented in Fig. 12(f), the largest increase in pressure coefficient at the vortex center, from $-1.40$ to $-0.13$, occurs at this high incidence when the gyroid tip portion is increased. At lower incidence angles Fig. 12(d, e), the minimum pressure coefficient is also substantially increased, but the effect is less pronounced with values rising from $-0.10$ to $-0.04$ at 4° and from $-0.50$ to $-0.10$ at 8°. This reduced impact at lower incidence angles can be attributed to the higher baseline (G0r) pressure coefficients; nevertheless, the gyroid tip still substantially raises the pressure coefficient and could help mitigate issues related to cavitation incipience.

### Effect on hydrodynamic performances

**Fig. 13:**
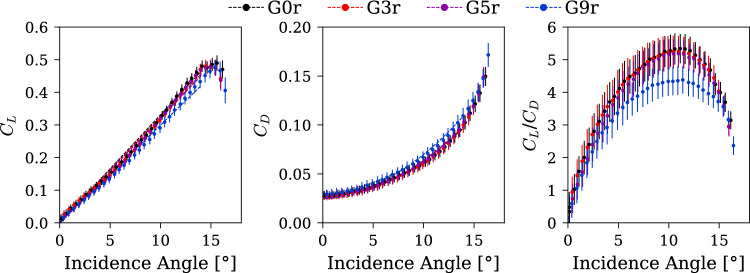
Force measurement and lift-to-drag ratio measured at $\mathrm{R}\mathrm{e}=9\times {10}^{5}$ with increasing gyroid tip portion, rough hydrofoil

The lift and drag coefficients, as well as the lift-to-drag ratio, are presented in Fig. 13 as a function of the incidence angle. We observe that the hydrofoil with the G0r insert exhibits the best performance. However, the G3r and G5r inserts are very close, lying within 1/10th of the standard deviation range. From the trend lines it seems that these inserts provide similar drag characteristics, with a very slight loss in lift coefficient. In contrast, the G9r insert provides substantially lower performance, which can be attributed to both reduced lift and increased drag. The tip vortex mitigation using a gyroid insert becomes a trade-off with performance, with 5% gyroid offering the best compromise. The decreased circulation observed in Fig. 12 for the G5r and G9r inserts does not consistently translate to a reduction in lift. We believe that the gyroid diffuses the concentrated vorticity into a broader wake, thereby reducing the measured circulation while preserving the total bound circulation and lift for the G5r insert. In contrast, the lift reduction observed with the G9r insert likely occurs because the permeable region extends beyond the primary tip vortex formation zone. Consequently, the permeable structure no longer operates exclusively within the vortex roll-up region but also alters the flow topology further inboard along the span.

### Effect of permeability

To evaluate the impact of permeability, a 5% non-permeable gyroid structure was also tested, blocking the flow through the gyroid tip and thus preserving only its surface texture. As presented in Figs. 7 and 9, the non-permeable G5Cr insert has a significantly higher tangential velocity and smaller core radius compared to the G5r insert. This results in a minimum core pressure even lower than the 3% insert as shown in Fig. 12. This demonstrates that the permeability of the insert is the key factor in reducing the tangential velocity. A slight increase in vortex core pressure is still observed with the 5% non-permeable insert compared to the 0% insert which may be attributed to increased tip roughness, which is known to influence vortex roll-up (Asnaghi et al. 2020).

### Effect of roughness

**Fig. 14:**
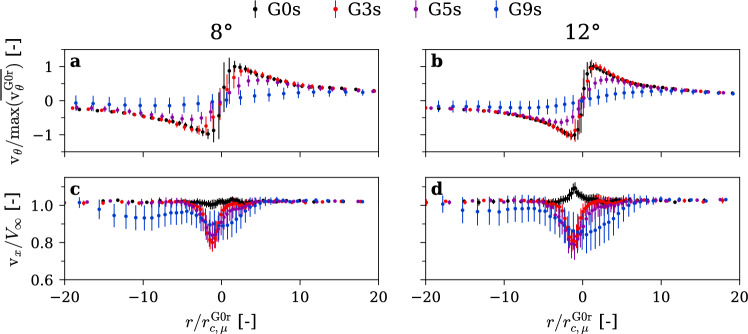
Mean and standard deviation of tangential (**a**, **b**) and streamwise (**c**, **d**) velocity profiles along the radial axis for increasing gyroid tip portion (0%, 3%, 5%, 9%) at incidence angles of 8°(**a**, **c**) and 12°(**b**, **d**). $\mathrm{R}\mathrm{e}=9\times {10}^{5}$, smooth hydrofoil

Figure 14 presents the mean and standard deviation of the tangential and axial velocities measured for the smooth hydrofoil. For the tangential velocities at incidence angles of 8° and 12°, we can see a very similar trend to the rough hydrofoil. Again, by increasing the gyroid portion at the foil tip, we greatly reduce the maximum tangential velocity and increase the vortex core radius. Outside the vortex core region, the velocity profiles are very similar, suggesting here also that the vortex circulation remains unchanged.

The axial velocity profile exhibits a jet-like behavior for the plain hydrofoil with smooth leading edge (G0S), consistent with Dreyer (2015). However, with the gyroid inserts G3S, G5S, and G9S, a wake-like behavior is observed, resulting from the surface roughness created by the gyroid surface. As the gyroid tip portion increases, the axial velocity deficit is again spread over a larger vortex core, leading to a slight reduction in minimum velocity, resulting in an overall similar velocity deficit.

## Conclusion

In this study, we demonstrated the exceptional potential of gyroid structures for tip vortex flow control. Acting as a *passive flow diverter* at the hydrofoil tip, this highly tortuous and permeable structure redistributes the tip leakage flow, substantially reducing vortex tangential velocity and increasing vortex core size, thereby significantly mitigating cavitation risks. The key findings are summarized as follows:

- Substantial tangential velocities reduction across all conditions: Tangential velocities were greatly reduced and vortex core sizes were significantly increased in all tested conditions. At 12° incidence, the 9% gyroid insert reduced peak tangential velocity by a factor of 3.2 alongside a sixfold increase in vortex core radius. These results were consistent for both rough and smooth hydrofoils’ leading edges.
- *Significant* cavitation risk reduction: The minimum pressure coefficient at the vortex core was substantially increased, greatly reducing cavitation risks. At 12° incidence, *C*_p,min_ was increased from − 1.4 to − 0.1. With the 5% gyroid insert, the foil transitioned from leading-edge cavitation to supercavitation without any visible tip vortex cavitation.
- Vorticity diffusion mechanism: As the gyroid portion increased, total vortex strength remained constant, indicating diffusion of vorticity into a larger vortex core. Both the vortex shape parameter and vortex wandering were modified with increasing gyroid portion, revealing subtle changes in the underlying flow patterns.
- Preservation of hydrodynamic performance: The hydrofoil’s lift and drag characteristics remained essentially unchanged with gyroid spans up to 5% at both low and high angles of attack. However, a further increase in the gyroid portion adversely affected both lift and drag, reducing hydrodynamic efficiency.
- Permeability-driven mechanism: The observed effects are predominantly related to structural permeability. When acting as a permeable flow diverter, the gyroid insert effectively alters the tip flow topology and promotes vorticity redistribution. In contrast, inserts with blocked permeability provide only surface roughness effects, leading to marginal tangential velocity reductions and negligible cavitation risk improvement.

These findings establish gyroid structures as a promising passive flow control device for mitigating tip vortex cavitation while maintaining hydrodynamic efficiency, offering practical applications for marine propellers, hydrofoils, and turbomachinery.

## Data Availability

Data generated during the current study are available from the corresponding author upon reasonable request.
